# Enhancement of fast scan cyclic voltammetry detection of dopamine with tryptophan-modified electrodes

**DOI:** 10.1371/journal.pone.0235407

**Published:** 2020-07-10

**Authors:** Sarah E. Davis, Andrew L. Korich, Eric S. Ramsson

**Affiliations:** Grand Valley State University, Allendale Charter Twp., Allendale, MI, United States of America; Nathan S Kline Institute, UNITED STATES

## Abstract

Fast scan cyclic voltammetry (FSCV) allows for real -time analysis of phasic neurotransmitter levels. Tryptophan (TRP) is an aromatic amino acid responsible for facilitating electron transfer kinetics in oxidoreductase enzymes. Previous work with TRP-modified electrodes showed increased sensitivity for cyclic voltammetry detection of dopamine (DA) when used with slower scan rates (0.05 V/s). Here, we outline an *in vitro* proof of concept for TRP-modified electrodes in FSCV detection of DA, and decreased sensitivity for ascorbic acid (AA). TRP-modified electrodes had a limit of detection (LOD) for DA of 2.480 ± 0.343 nM compared to 8.348 ± 0.405 nM for an uncoated electrode. Selectivity for DA/ascorbic acid (AA) was 1.107 ± 0.3643 for uncoated and 15.57 ± 4.184 for TRP-modified electrodes. Additionally, these TRP-modified electrodes demonstrated reproducibility when exposed to extended cycling. TRP-modified electrodes will provide an effective modification to increase sensitivity for DA.

## Introduction

Dopamine (DA) is a catecholamine neurotransmitter that plays a critical role in the development and treatment of neurodegenerative diseases including Alzheimer’s and Parkinson’s disease [1, 2]. Both diseases lead to dopaminergic cell death and decreased extracellular levels of DA [3, 4]. Micro-dialysis and fast scan cyclic voltammetry (FSCV) are two widely used tools used for extracellular neurotransmitter analysis [5]. Micro-dialysis involves extraction of cerebrospinal fluid (CSF) and subsequent quantitative analysis of neurotransmitter levels [6]. This tool is sufficiently sensitive for detecting low levels of DA, with the limit of detection (LOD) having been reported as low as 5 pg/mL, approx. 0.0326 nM [7]. The LOD for FSCV detection has been reported near 15 ± 1 nM when using T-650 carbon fibers [8]. Whereas micro-dialysis offers an advantage in DA sensitivity, FSCV outperforms in temporal resolution by allowing for sub-second detection of DA [5, 9]. Improving the sensitivity of FSCV will aid in understanding the pathology of central nervous system (CNS) diseases which exhibit decreased extracellular levels of DA.

The most conserved amino acid in nature, tryptophan (TRP), has been found to be highly conserved in more than half the oxidoreductases in the Enzyme Data Bank of the Swiss Institute of Bioinformatics [10]. Previously, the enzyme Cytochrome C was immobilized on microelectrodes and demonstrated an enhancement in electron transfer during cyclic voltammetry analysis of redoxable analytes [11]. Mutation studies in prokaryotic [6–4] photolyase and Cytochrome C found that TRP residues arranged in a chain were responsible for electron transfer kinetics in enzymatic reactions [12, 13]. Based on the role of TRP in nature as a facilitator in redox electron transfer kinetics, we hypothesize that immobilized TRP on the surface of carbon microelectrodes will increase the electron transfer kinetics of the DA redox reaction, thus increasing the peak oxidative current detected, thereby improving the LOD of FSCV detection of DA.

In addition to the role of TRP in nature, TRP-modified and 5-hydroxytryptophan (5-HTP) modified electrodes were investigated previously using cyclic voltammetry at slower scan rates (scan rate = 0.05 V/s) and showed an increase in sensitivity for DA [14]. Although 5-HTP electrodes showed the greatest increase in sensitivity for DA, they also showed an increase in sensitivity for uric acid (UA), ascorbic acid (AA), and serotonin (5-HT) [15]. Since our focus here is to detect DA, using a 5-HT modified electrode at fast scan rates (scan rate = 400 V/s) could potentially result in increased sensitivity for off-target metabolites. Therefore, we focused on investigating the performance of TRP-modified electrodes for DA detection.

Here, we outline an *in vitro* proof of concept for TRP-modified carbon fiber microelectrodes for DA detection. We tested TRP-modified electrodes in their performance for increased sensitivity for DA at fast scan rates and selectivity for DA compared to AA. Optimal parameters for electrodeposition of TRP on the electrodes were investigated, in addition to the potential mechanism of increased sensitivity. Our data suggest that TRP-modified electrodes are a suitable modification for increased FSCV sensitivity for DA.

## Methods

### Electrode preparation

Microelectrodes were fabricated with 7 μm diameter carbon fiber (T-650, Cytec Engineering, Woodland Park, NJ) and sealed with paraffin [16]. Electrodes were cycled prior to all experiments in a potential window of -0.4–1.0 V for 15 minutes at a frequency of 60 Hz, then immediately after for 5 minutes at a frequency of 10 Hz, in artificial CSF (aCSF, 125 mM NaCl, 4 mM KCl, 1.3 mM CaCl_2_, 1 mM MgCl_2_, 0.66 mM NaH_2_PO_4_, 2 mM Na_2_HPO_4_, 1 mM glucose; pH 7.4). All experiments were performed vs. Ag/AgCl using the Demon Voltammetry system [17]. Electrodes were cut to approximately 100 μm and measured with ToupView software (Amscope, San Diego, CA). Exact measurements of electrode length were used to calculate electrode surface area (unless otherwise indicated) and normalize peak oxidation current for electrode size (i_p_ density). All reagents were purchased from Thermo Fisher Scientific or Sigma Aldrich.

### TRP deposition

1mM and 10 mM TRP was prepared in phosphate buffer saline (PBS, 140 mM NaCl, 3 mM KCl, 10 mM NaH_2_PO_4_; pH 7.4). Electrodes were cycled prior to TRP deposition for 10 minutes at a frequency of 10 Hz in PBS before TRP deposition during paired Control vs. Modified tests. TRP deposition was performed with a scan rate of 0.02 V/s and a potential window of -1.7–1.8 V, for 3 complete cycles [14, 15]. Cyclic voltammetry for experiments with a scan rate ≤ 2 V/s were performed with a 1200B model CH Instruments potentiostat (CH Instruments, Inc. 3700 Tennison Hill Drive Austin, TX). Cyclic voltammetry for experiments with a scan rate > 2 V/s were performed vs. an Ag/AgCl reference using a ChemClamp potentiostat (Dagan, Minneapolis, MN).

### DA and AA detection

DA experiments were performed *in vitro* using a pipette-based calibration configuration as previously described [18]. Concentrations of 50 nM, 100 nM, 200 nM, 500 nM, and 1 μM DA were used for the standard curve. 1 μM DA and 200 μM AA were used for testing selectivity for DA vs. AA. All experiments were performed with a scan rate of 400 V/s in a potential window of -0.4–1.0 V vs. an Ag/AgCl reference using a ChemClamp potentiostat (Dagan, Minneapolis, MN). Scan window had a peak of 1.0 V instead of the traditional 1.3 V to minimize change in electrode size which itself can contribute to increased sensitivity [19]. Background subtraction was performed using Demon Voltammetry software for all experiments with scan rate > 2 V/s [17]. DA and AA collections were performed at 10 Hz for 30 seconds to prevent background fluctuation.

### Statistical analysis

All statistical analyses were performed using GraphPad Prism 8. Statistical tests used for each figure are mentioned in their respective discussion sections. A p value < 0.05 was used to determine significance.

## Results

### Modified electrodes have increased sensitivity for DA

**Fig 1:**
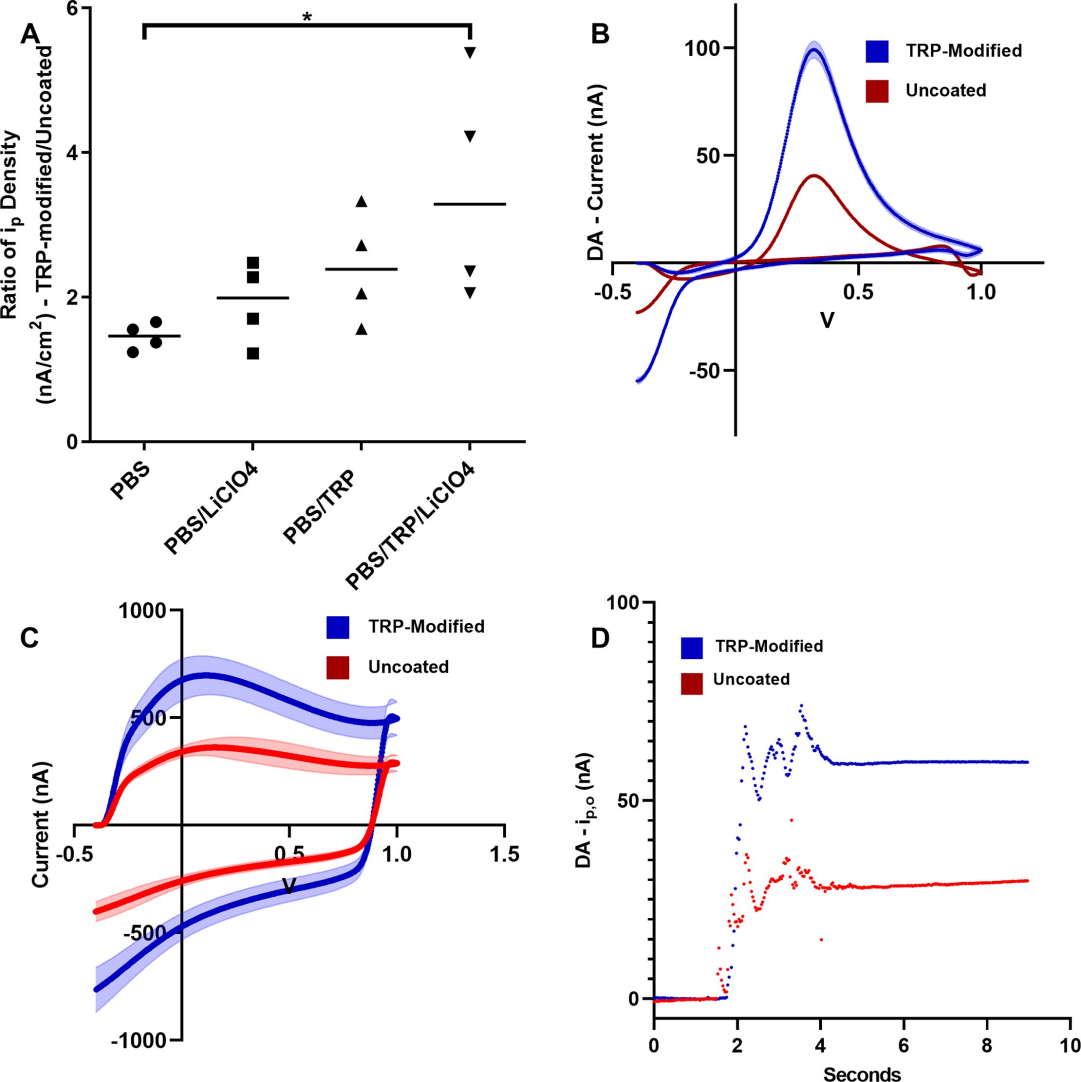
A. Electrodes were treated with either PBS, PBS/LiClO_4_, PBS/TRP, or PBS/TRP/LiClO_4_. Ratio of peak current density (i_p_, nA/cm^2^) for oxidation of DA using a TRP-modified/uncoated electrode was used for quantification of DA sensitivity. 1 μM DA was used for all experiments. Significant differences are indicated by * (p ≤ 0.05; n = 4 per group). Kruskal-Wallis analysis indicated a significant difference (p = 0.0354). Post hoc analysis showed a significant difference between PBS vs. PBS/TRP/LiClO_4_ (p = 0.0451). B. Background subtracted cyclic voltammograms for uncoated and TRP-modified electrodes are shown for 1 μM DA detection (uncoated = red, TRP-modified = blue). Collections were performed in a potential window of -0.4–1.0 V with a scan rate of 400 V/s, vs. Ag/AgCl. Peak oxidative current here is shown as positive nA. Error is shown as the shaded area around the voltammogram. C. Background cyclic voltammograms collected in aCSF for uncoated and TRP-modified electrodes are shown above, n = 4. D. Current time traces for 1 μM DA collection (control = red, modified = blue), n = 4.

Increased sensitivity for DA with modified electrodes was investigated with 30 second collections of 1 μM DA (Fig 1A). Lin and Li [15] used PBS/TRP/LiClO_4_ to modify electrodes for increased sensitivity, and to determine if increased sensitivity was due to TRP or LiClO_4_, electrodes were modified with the following conditions: PBS, PBS/LiClO_4_, PBS/TRP, and PBS/TRP/LiClO_4._ For all data presented after Fig 1A, TRP-modified refers to electrodes treated with PBS/TRP/LiClO_4._ Electrodes were modified using a potential window of -1.7–1.8 V. Electrodes exposed to potentials greater than 1.0 V have been shown to oxidize and renew their surface [19]. The oxidation of the surface in this potential window leads to an increased level of sensitivity. To determine if an increase in sensitivity is due to surface oxidation or TRP we compared the ratio of current density (i_p_/electrode size) for the TRP-modified/uncoated electrodes to establish if the change in sensitivity observed for the treatment groups was different than the expected change in sensitivity from surface oxidation at exposure to potentials > 1.0 V. Note, all i_p_ densities used in this study are for the i_p_ of oxidation. Brown-Forsythe test showed heterogeneity among variances (p = 0.0038), therefore Kruskal-Wallis was used as a nonparametric alternative to the one-way ANOVA test. Kruskal-Wallis test was significant (p = 0.0354), and further post hoc analysis (Dunn’s multiple tests) revealed a significant difference between PBS vs. PBS/TRP/LiClO_4_ (p = 0.0451). There was no significant difference between the PBS vs. PBS/TRP and PBS/TRP vs. PBS/TRP/LiClO_4_ groups. Moving forward all TRP-modification was performed using PBS/TRP/LiClO_4_.

Cyclic voltammograms for 1 μM DA are shown illustrating the increased oxidation peak for DA when using uncoated vs. TRP-modified electrodes (Fig 1B). TRP-modified electrodes had an increased background current (Fig 1C) and a longer rise time for saturation of the electrode with DA (Fig 1D). ΔE_p_ is not significantly different between uncoated and TRP-modified electrodes (Table 1).

**Table 1:** Characterization of TRP-modified electrodes for detection of DA.

Electrode Type	DA LOD (nM)	DA Sensitivity (nA/μM)	DA/AA Selectivity (nA, 200 μM AA, 1 μM DA)	E_p,o_ (V, 200 nM DA)	E_p,r_ (V, 200 nM DA)	ΔE_p_ (V, 200 nM DA)
Uncoated	8.348 ± 0.405	12.802 ± 2.804	1.107 ± 0.3643	0.426 ± 0.026	-0.323 ± 0.046	0.749 ± 0.027
TRP-modified	2.480 ± 0.343	26.425 ± 5.690	15.57 ± 4.184	0.460 ± 0.030	-0.320 ± 0.026	0.7804 ± 0.031

### Optimization of TRP deposition parameters

**Fig 2:**
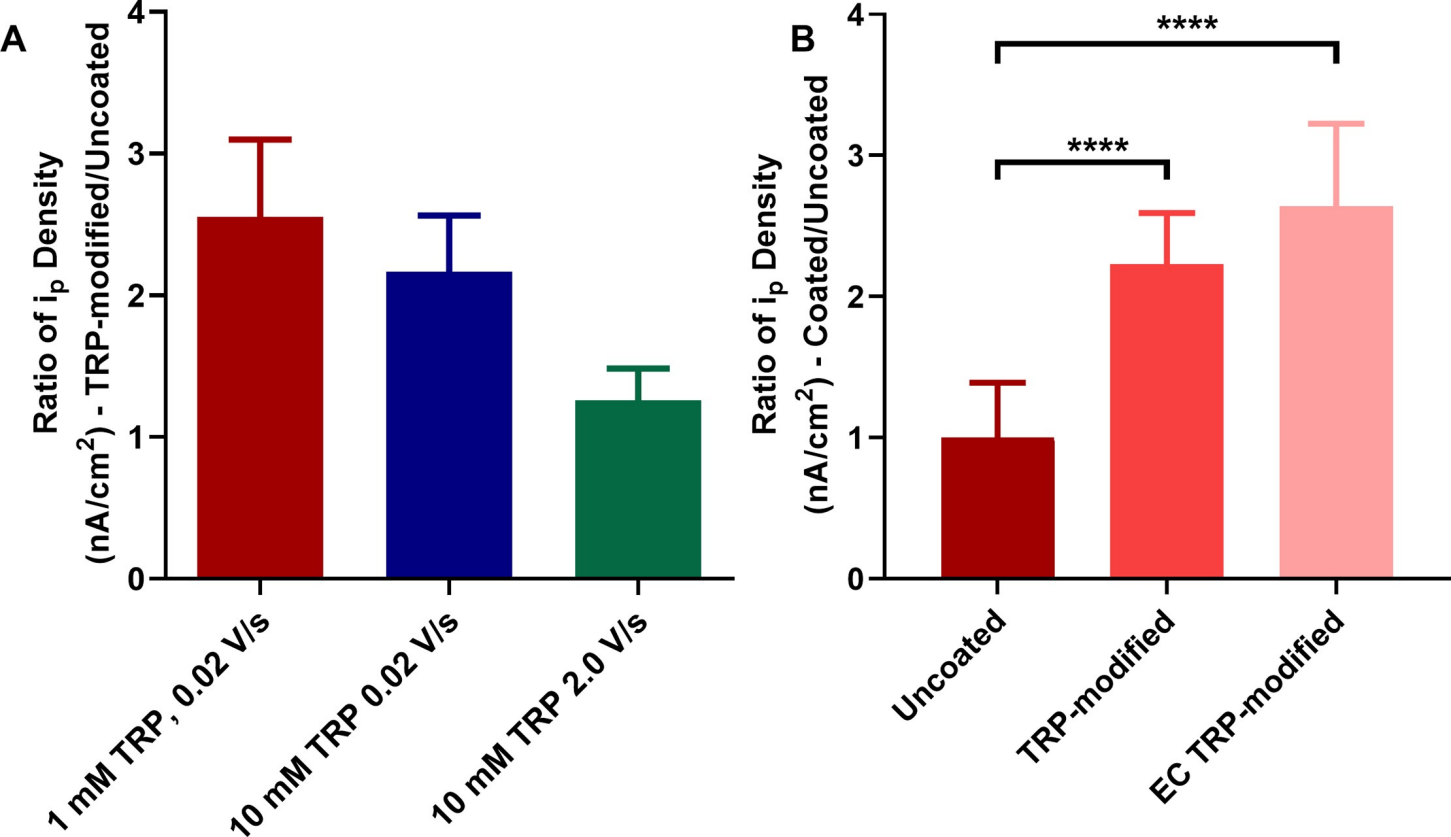
A. Ratio of i_p_ density for DA (TRP-modified/uncoated) using electrodes treated with either 1 mM TRP at 0.02V/s, 10 mM TRP at 0.02 V/s, or 10 mM TRP at 2.0 V/s is shown. Collections were performed for 1 μM DA for 30 seconds. One-way ANOVA analysis indicated no significant difference between means (p = 0.1279), n = 4 per group. B. Collections of 1 μM DA were performed using the uncoated electrode, TRP-modified electrode, and EC TRP-modified electrode. One-way ANOVA analysis indicated a significant difference between means (p < 0.001), n = 4 per group. Post hoc analysis (multiple comparisons, Tukey’s test); Uncoated vs. TRP-modified (p < 0.0001), Uncoated vs. EC TRP-modified (p < 0.001), TRP-modified vs. EC TRP-modified (p = 0.0835).

The parameters for TRP deposition of 1 mM TRP and a scan rate of 0.02 V/s were used as previously described [14]. We tested the effect of TRP concentration and scan rate of TRP deposition to improve the electrode performance for detection of DA. One-way ANOVA for the optimization of TRP concentration and scan rate parameters indicated no significant difference between groups (p = 0.1279, n = 4) (Fig 2A).

Further investigation showed the deposition of TRP onto the electrode surface is a diffusion-controlled reaction. Previously, cyclic voltammetry of TRP showed an irreversible oxidation of TRP in a diffusion-controlled manner [20]. We confirmed that TRP was oxidizing in a diffusion-controlled manner by plotting log(i_p_) vs. log(v) (scan rates = 0.02, 0.2, 2, 20, and 200 V/s) using Eq 1. for an irreversible diffusion-controlled reaction at a planar electrode, where (A); n: number of electrons; α: transfer coefficient; n_α_: number of electrons in charge transfer step; A: surface area (cm^2^); Do: diffusion coefficient (cm^2^/s), C: bulk concentration (mol/cm^3^); v: scan rate (V/s) [21]. Although our electrodes are cylindrical, diffusion at the electrode surface can be treated as a planar diffusion if the scan rate is sufficiently fast, and the electrode radius is sufficiently small compared to the electrode length [22]. Our electrodes were cut to approximately 100 μm and had a radius of 3.5 μm, allowing us to treat our electrode as planar.

$$i_{p}=(2.99*10^{5})n{\left(\alpha n_{\alpha }\right)}^{1/2}ACD^{1/2}v^{1/2}$$ Eq 1

The slope of the curve was 0.5042, indicating a diffusion-controlled reaction [23]. Deposition of TRP at 2.0 V/s was sufficient for providing an increase in DA sensitivity. This change was not significantly different from the increase in sensitivity from electrodes modified with parameters 1 mM TRP and 0.02V/s.

### Modified electrodes demonstrate reproducible increased sensitivity for DA

Next, we asked whether the increased sensitivity for DA using TRP-modified electrodes could be reproduced after exposing the electrode to repeated cycling. Repeated cycling mimics extended data collection experiments. Electrodes were modified with PBS/TRP/LiClO_4_ and then exposed to extended cycling (EC), which was performed as follows: scan rate of 400 V/s, potential window -0.4–1.0 V, freq. = 60 Hz for 10 minutes, then freq. = 10 Hz for 5 minutes. DA collections were performed before TRP-modification, after TRP-modification, and after EC. One-way ANOVA for the test of reproducibility with TRP-modified electrodes after EC was significant (p value = <0.0001, n = 4) (Fig 2B). Post Hoc analysis (multiple comparisons, Tukey’s test) showed a significant increase in sensitivity for Control vs TRP-modified (p <0.0001), Control vs. EC TRP-modified (p < 0.0001) and TRP-modified vs. EC TRP-modified (p = 0.0392).

### Standard curve and LOD for DA detection

**Fig 3:**
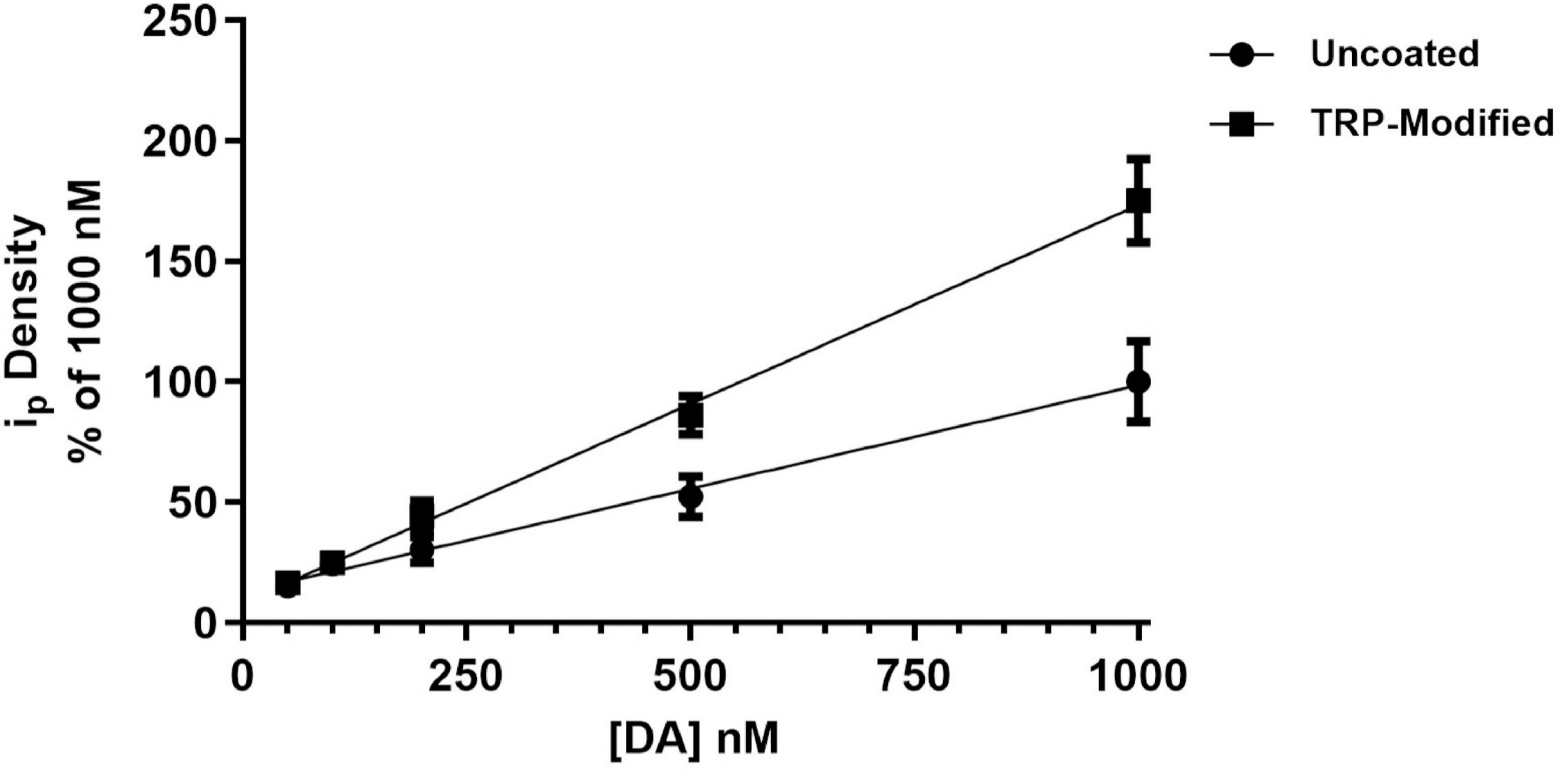
I_p_ density for DA using uncoated and TRP-modified electrodes was plotted vs. known DA standards (50 nM, 100 nM, 200 nM, 500 nM, 1 μM), n = 5 per group normalized to uncoated 1000 nM mean response. Slopes for the uncoated and TRP-modified curves are 0.0860 ± 0.0038 (nA·Q^-1^·nM^-1^), and 0.1649 ± 0.0042 (nA·Q^-1^·nM^-1^), respectively. ANCOVA showed that the slopes were significantly different (p < 0.0001).

To investigate the scope of increased DA sensitivity, a standard curve was created using [DA] = 50, 100, 200, 500, and 1000 nM. Data is shown as a % of the current for 1000 nM uncoated electrode. Slopes for the uncoated and TRP-modified electrode were 0.0860 ± 0.0038 (nA·Q^-1^·nM^-1^), and 0.1649 ± 0.0042 (nA·Q^-1^·nM^-1^), respectively (Fig 3). The slope of the curve for the TRP-modified electrodes was approximately 1.92 times greater than that for the uncoated electrode. ANCOVA indicates the slopes were significantly different (p < 0.0001).

The limit of detection (LOD) was determined using the concentration of DA at which the signal:noise ratio is 3 [24]. The absolute value of the background subtracted current during the sweep from 0 V to 1.0 V was used to quantify noise. The LOD for the unmodified electrode was 8.348 ± 0.405 nM, and for TRP-modified was 2.480 ± 0.343 nM (Table 1). The LOD for the uncoated electrode is slightly lower than what has been reported for uncoated T-650 carbon fibers [8].

### Selectivity for DA vs. AA

**Fig 4:**
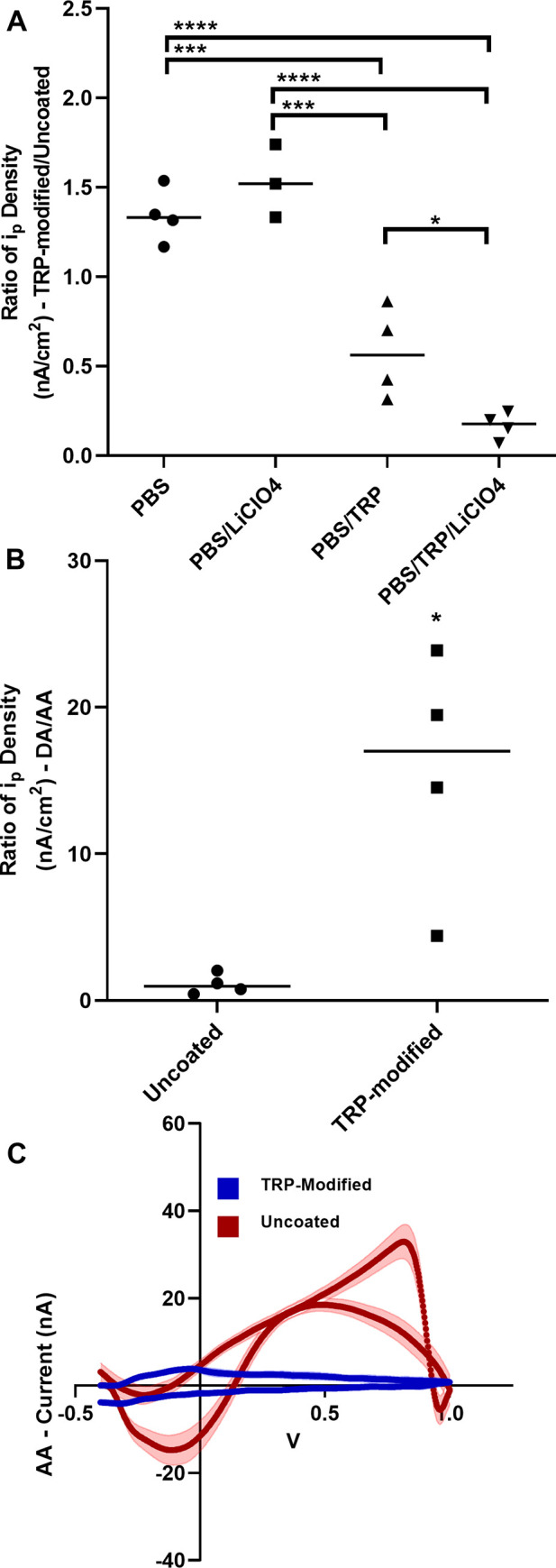
A. Electrodes were treated with either PBS, PBS/LiClO_4_, PBS/TRP, or PBS/TRP/LiClO_4_. Ratio of peak current density (i_p_, nA/cm^2^) for oxidation of AA using a TRP-modified/uncoated electrode was used for quantification of AA sensitivity. 200 μM AA was used for all experiments. Significant differences after post hoc analysis (Multiple comparisons, Tukey’s test) between treatment groups are indicated (n = 4, per group). B. Ratio of i_p_ density for DA/AA for uncoated and TRP-modified electrodes detecting 1 μM DA and 200 μM AA. Unpaired t test with Welch’s correction showed a significant difference between groups, as indicated (p = 0.0402, n = 4 per group). C. Cyclic voltammograms shown above for 200 μM AA detection (uncoated = red, TRP-modified = blue, for 20 cycles).

The objective of electrode modification was to increase sensitivity for DA; however, DA is not the only metabolite present in a dopaminergic synapse which oxidizes and reduces in the -0.4–1.0 V potential window, therefore it is necessary to investigate TRP-modified electrode detection of the competing metabolite AA [25, 26]. AA is present in the dopaminergic synapse at high concentrations ranging from 200–400 μM [27]. As with the sensitivity tests for DA, electrodes were used to detect 200 uM AA before and after modification with either PBS, PBS/LiClO_4_, PBS/TRP, or PBS/TRP/LiClO_4_. Ratios of i_p_ density TRP-modified/uncoated were used to determine if increased changes in sensitivity are due to TRP and not surface oxidation. One-way ANOVA for AA sensitivity was significant (p v < 0.0001). Results for post hoc analysis (multiple comparisons, Tukey’s test) were as follows: PBS vs. PBS/TRP (p = 0.0004), PBS vs. PBS/TRP/LiClO_4_ (p < 0.0001), PBS/LiClO_4_ vs PBS/TRP (p = 0.0001), PBS/LiClO4 vs. PBS/LiClO_4_/TRP (p < 0.0001), PBS/TRP vs. PBS/TRP/LiClO_4_ (p = 0.0366). PBS vs PBS/LiClO_4_ was not significant (p = 0.542) (Fig 4A).

Additionally, we compared the ratio of i_p_ density for DA/AA for control electrodes and modified electrodes (Fig 4B). There was a significant difference between the uncoated and TRP-modified electrodes for ratio of DA/AA sensitivity (Unpaired t- test with Welch’s correction, p value = 0.0402). The ratios of DA/AA i_p_ density were 1.107 ± 0.3643 and 15.57 ± 4.184 for uncoated and TRP-modified electrodes, respectively. Background subtracted cyclic voltammograms for AA using uncoated and TRP-modified electrodes are shown to illustrate changes AA in sensitivity for different electrodes (Fig 4C).

## Discussion

TRP-modified electrodes have enhanced sensitivity for DA that is reproducible, and decreased sensitivity for AA. The proposed mechanism behind these changes is discussed further in the following sections. The LOD for DA detection was improved, from 8.348 ± 0.405 nM (uncoated) to 2.480 ± 0.343 nM (TRP-modified). The uncoated electrode had a lower LOD than previously reported for T-650 fibers (15 ± 1 nM) [8]. Our electrodes were cycled for 15 minutes at 60 Hz then 5 minutes at 10 Hz prior to use to clean the surface of the electrode, however pretreatment of the electrodes from Puthongkham et. al 2018 was not discussed. Therefore, we cannot confirm if the difference in LOD for unmodified electrodes was due to varying cleaning parameters, which could possibly explain this observation.

### TRP modification and mechanism for selectivity

For TRP deposition we included LiClO_4_ in the deposition solution based on previous work by Li and colleagues [14] which modified electrodes with 5-HT and TRP for detection of DA and 5-HT. Justification for the use of LiClO_4_ in the TRP deposition solution was not provided. LiClO_4_ is a strong oxidizing reagent, therefore it is possible that is facilitating the oxidation reaction of TRP, thus immobilizing more TRP on the electrode surface [28].

Lin and Li [15] showed that 5-HT covalently binds to carbon fiber electrodes, which they confirmed with x-ray photoelectron spectroscopy (XPS). 5-HT is a primary amine, and the binding energy (399.4 eV) for the XPS data reported by Lin and Li [15] is in agreement with that of primary amines covalently attached to carbon fiber via electrochemical modification, which indicates a carbon–nitrogen (C-N) bond [29, 30]. It is well established that TRP irreversibly oxidizes when exposed to positive potentials, however there is no direct evidence that TRP covalently attaches to the electrode surface as a result of this irreversible oxidation [31, 32]. The 5-HT XPS data strongly indicates that TRP modifies the surface in the same manner, via a C-N bond. The only difference between 5-HT and TRP is the presence of a hydroxyl group on the indole group of the amino acid. The hydroxyl group lacks a nitrogen necessary for the C-N bond, therefore, it is possible that TRP can attach to the surface in the same manner as 5-HT. However, the hydroxyl group of 5-HT was necessary for the C-N to form, but there is evidence which shows that it is the amine group of primary amines, regardless of other R groups, is responsible for covalently binding to the surface [29, 30]. Additionally, our demonstration of reproducible increased sensitivity for DA and decreased sensitivity for AA, indirectly supports the TRP covalent modification to the carbon surface.

**Fig 5:**
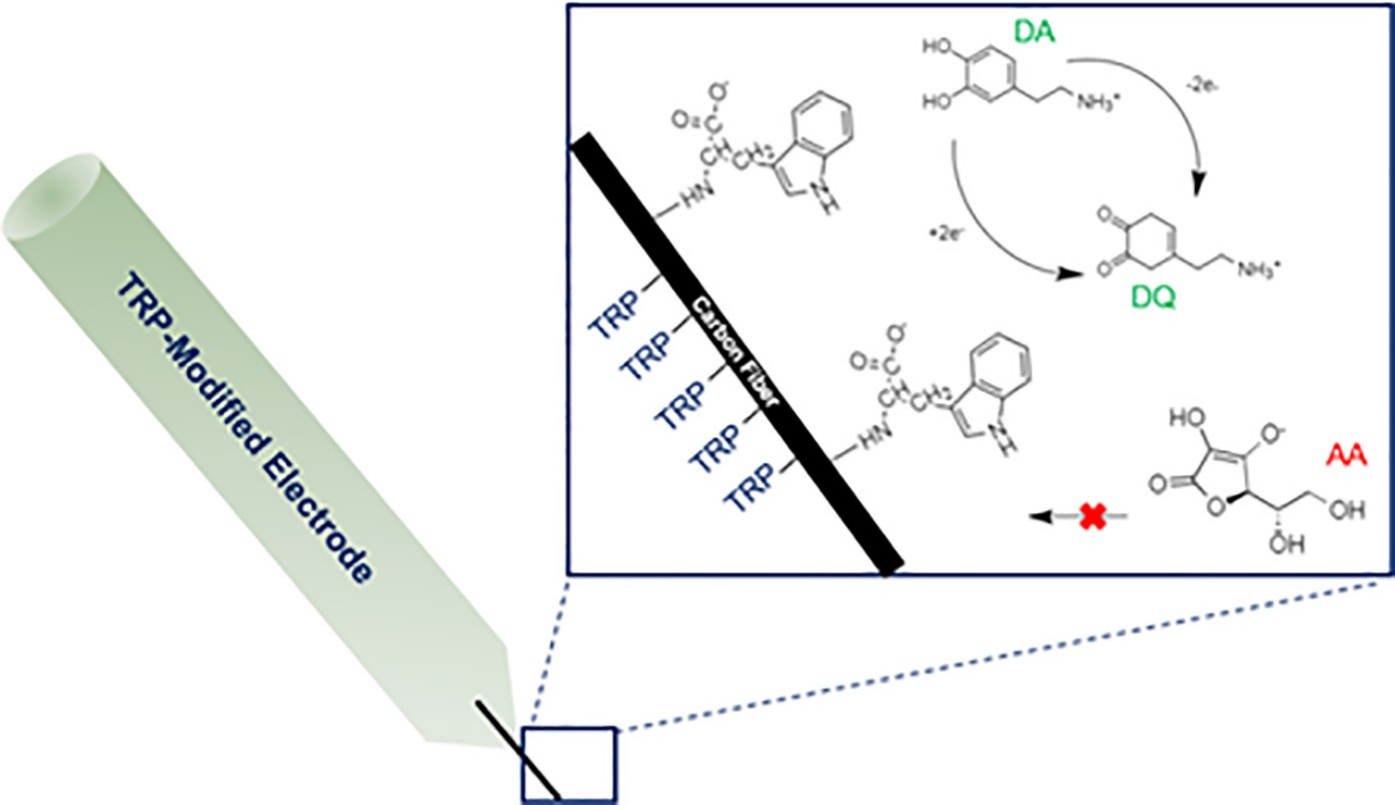
A proposed model for TRP-modification based on carbon–nitrogen binding at the amine group. AA is negatively charged and repelled by the negatively charged TRP surface, whereas positively charged DA is adsorbed.

At a physiological pH TRP is a zwitterion. If the 1° amine group of TRP is covalently binding to the surface of the electrode as we have just suggested, then the positive charge of the zwitterion will be neutralized, yielding a negative charge on the electrode surface. Reports modifying the surface of microelectrodes have suggested a change in the sign of the charge of the electrode surface has helped to improve selectivity for DA over competing metabolites [25]. DA is a positively charged metabolite at a physiological pH of 7.4, whereas AA is negatively charged. The surface of the TRP- modified electrode may have a greater net negative charge, thus repelling AA and attracting DA. A model for TRP-modification and AA vs. DA detection is shown in Fig 5.

### DA vs. AA sensitivity

Based on the role of TRP in nature we hypothesized that the mechanism behind TRP’s increased sensitivity for DA was due to faster electron transfer kinetics, however there was no significant difference in ΔE_p_ between uncoated and TRP-modified electrodes. Current vs. time traces showed a longer rise time for DA detection, indicating slower mass transport. Although ΔE_p_ did not change it is still possible that there was an increase in electron transfer kinetics which is being masked by the slow transport of DA to the electrode surface [33]. Alternatively, the electrode size may be larger due to TRP-modification, thus creating more reactive sites for DA to adsorb to. The background current was larger for TRP-modified electrodes therefore it is more likely that increased sensitivity for DA is due to an increase in reactive sites.

Electrodes treated with PBS/TRP and PBS/TRP/LiClO4 both significantly decrease sensitivity for AA, however only electrodes treated with PBS/TRP/LiClO4 significantly increased sensitivity for DA, although, there was a trend for increased sensitivity for DA when using PBS/TRP. Deposition without LiClO_4_ may not provide a sufficient amount of TRP to the surface to significantly increase the number of reactive sites for DA. Conversely, AA does not need to diffuse to the surface to react, therefore less TRP is needed to see a difference in sensitivity. This would explain why PBS/TRP deposition was sufficient to significantly decrease sensitivity for AA, but not sufficient to significantly increase sensitivity for DA.

## Conclusion

In this study we outline an *in vitro* proof of concept for the use of electrodes modified with TRP and LiClO_4_. TRP-modified electrodes had an increase in sensitivity for DA and a decrease in sensitivity for AA. In addition, TRP-modified electrodes demonstrated reproducible increased sensitivity for detection of DA after exposure to repeated cycling in a potential window of -0.4–1.0 V, and an improved LOD. The mechanism for changes in sensitivity for DA and AA is not fully understood, however we suggest that TRP increases the surface area of the electrode, thereby creating more reactive sites for DA to adsorb to. Thus, this data supports the use of TRP for FSCV electrode modification.

## Abbreviations

FSCV: Fast Scan Cyclic Voltammetry
TRP: tryptophan
DA: dopamine
CNS: central nervous system
UA: uric acid
AA: ascorbic acid
5-HT: 5-hydroxytryphohan
PBS: phosphate buffer saline
aCSF: artificial cerebral spinal fluid
LOD: limit of detection
EC: extended cycling
XPS: x-ray photoelectron spectroscopy
